# Supplementary material for: Serum Vitamin D Affected Type 2 Diabetes though Altering Lipid Profile and Modified the Effects of Testosterone on Diabetes Status
Source: Nutrients. 2020 Dec 30;13(1):90. doi: 10.3390/nu13010090 (PMC7823697; doi:10.3390/nu13010090)
Supplement: Supplementary file 1 [file nutrients-13-00090-s001.pdf]

Supplement Table 1 Associations of serum 25(OH)D_3, 25(OH)D_2, and total 25(OH)D levels with IFG and T2DM

	Logistic regression, *OR*s (95%*CI*s)				
	Cases/Controls	Median (range)	Model 1	Model 2	Model 3
**IFG**					
25(OH)D_3					
Continuous	913/897	22.59 (5.30- 67.06)	0.98 (0.97, 0.99)*	0.98 (0.97, 0.99)*	0.99 (0.97, 1.00)*
Q1	251/186	16.00 (5.30- 18.55)	Reference	Reference	Reference
Q2	234/240	20.51 (18.59- 22.62)	0.72 (0.56, 0.94)*	0.73 (0.56, 0.95)*	0.77 (0.59, 1.01)
Q3	222/236	24.97 (22.64- 27.61)	0.70 (0.54, 0.91)*	0.71 (0.54, 0.92)*	0.78 (0.59, 1.03)
Q4	206/235	31.99 (27.63- 67.06)	0.65 (0.50, 0.85)*	0.66 (0.50, 0.86)*	0.75 (0.57, 1.00)*
P for trend			0.002	0.003	<0.001
25(OH)D_2					
Continuous	913/897	6.98 (3.15- 37.61)	0.85 (0.82, 0.88)*	0.84 (0.81, 0.87)*	0.85 (0.82, 0.88)*
Q1	242/153	4.86 (3.15- 5.45)	Reference	Reference	Reference
Q2	260/172	6.09 (5.47- 6.71)	0.96 (0.72, 1.26)	0.954 (0.721, 1.263)	0.96 (0.72, 1.29)
Q3	239/236	7.45 (6.73- 8.44)	0.64 (0.49, 0.84)*	0.631 (0.480, 0.829)*	0.63 (0.48, 0.84)*
Q4	172/336	10.56 (8.46- 37.61)	0.32 (0.25, 0.43)*	0.318 (0.241, 0.420)*	0.33 (0.25, 0.44)*
P for trend			<0.001	<0.001	<0.001
25(OH)D					
Continuous	913/897	30.06 (8.45- 81.21)	0.96 (0.95, 0.97)*	0.96 (0.95, 0.97)*	0.97 (0.96, 0.98)*
Q1	256/181	22.56 (8.45- 25.25)	Reference	Reference	Reference
Q2	250/211	27.84 (25.25- 30.02)	0.84 (0.64, 1.09)	0.84 (0.65, 1.10)	0.87 (0.67, 1.15)
Q3	251/255	32.92 (30.03- 36.66)	0.70 (0.54, 0.90)*	0.70 (0.54, 0.91)*	0.76 (0.58, 0.99)*
Q4	156/250	41.16 (36.67- 81.21)	0.44 (0.34, 0.58)*	0.44 (0.33, 0.58)*	0.50 (0.37, 0.66)*
P for trend			<0.001	<0.001	<0.001
**T2DM**					
25(OH)D_3					
Continuous	849/897	22.93 (5.30- 67.06)	0.99(0.98, 1.00)	0.99 (0.98, 1.00)	0.99 (0.98, 1.01)
Q1	226/186	15.96 (5.30- 18.55)	Reference	Reference	Reference
Q2	190/240	20.68 (18.59- 22.62)	0.65 (0.50, 0.86)*	0.66 (0.50, 0.87)*	0.68 (0.50, 0.92)*
Q3	207/236	25.18 (22.64- 27.61)	0.72 (0.55, 0.95)*	0.72 (0.55, 0.95)*	0.71 (0.52, 0.95)*
Q4	226/235	31.99 (27.63- 67.06)	0.79 (0.61, 1.03)	0.80 (0.61, 1.04)	0.86 (0.64, 1.17)
P for trend			0.193	0.191	0.421
25(OH)D_2					
Continuous	849/897	6.87 (3.15- 37.61)	0.85 (0.81, 0.88)*	0.84 (0.81, 0.88)*	0.84 (0.81, 0.88)*
Q1	256/153	4.86 (3.15- 5.46)	Reference	Reference	Reference
Q2	245/172	6.09 (5.47- 6.71)	0.85 (0.64, 1.13)	0.84 (0.64, 1.12)	0.93 (0.68, 1.26)
Q3	191/236	7.50 (6.73- 8.44)	0.48 (0.37, 0.64)*	0.47 (0.36, 0.63)*	0.47 (0.35, 0.64)*

Q4	157/336	10.56 (8.48- 37.61)	0.28 (0.21, 0.37)*	0.28 (0.21, 0.36)*	0.27 (0.20, 0.37)*
P for trend			<0.001	<0.001	<0.001
25(OH)D					
Continuous	849/897	30.52 (8.45- 81.21)	0.97 (0.96, 0.98)*	0.97 (0.96, 0.98)*	0.97 (0.96, 0.99)*
Q1	226/181	22.45 (8.45- 25.25)	Reference	Reference	Reference
Q2	205/211	27.76 (25.25- 30.02)	0.78 (0.59, 1.02)	0.78 (0.59, 1.03)	0.77 (0.57, 1.04)
Q3	240/255	32.75 (30.03- 36.66)	0.75 (0.58, 0.98)*	0.76 (0.58, 0.99)*	0.74 (0.55, 0.99)*
Q4	178/250	41.47 (36.67- 81.21)	0.57 (0.43, 0.75)*	0.57 (0.43, 0.75)*	0.61 (0.45, 0.83)*
P for trend			<0.001	<0.001	0.002

Model 1: no adjust. Model 2: adjusted for smoking status, alcohol intake, physical activity, average monthly individual income, level of education. Model 3: model 2+ BMI, SBP, PP, TC, TG, HDL-C, LDL-C and family history of T2DM. *$P<0.05$. Abbreviations: IFG, impaired fasting glucose; T2DM, type 2 diabetes mellitus; *OR*, odds ratio; 95%*CI*: 95% confidence interval; Q1, the first/ lowest quartile; Q2, the second quartile; Q3, the third quartile; Q4, the fourth/highest quartile.

Supplement Table 2 The adjusted *β* coefficients (95% *CI*s) in markers of glucose metabolism associated with serum 25(OH)D_3, 25(OH)D_2, 25(OH)D concentrations

Variables	Adjusted *β* coefficients (95% *CI*s)				
	FPG	HbA1c	INS	Ln-HOMA2-IR	Ln-HOMA2-β
25(OH)D_3					
Continuous	0.01 (-0.00, 0.03)	0.00 (-0.01, 0.01)	-0.04 (-0.07, -0.01)*	-0.00 (-0.01, -0.00)*	-0.00 (-0.01, -0.00)*
Q1	Reference	Reference	Reference	Reference	Reference
Q2	-0.07 (-0.34, 0.19)	-0.09 (-0.25, 0.07)	0.06 (-0.53, 0.66)	0.01 (-0.04, 0.05)	0.04 (-0.03, 0.10)
Q3	0.17 (-0.09, 0.44)	0.01 (-0.16, 0.17)	-0.25 (-0.84, 0.34)	-0.00 (-0.05, 0.04)	-0.03 (-0.09, 0.04)
Q4	0.22 (-0.05, 0.49)	-0.00 (-0.17, 0.17)	-0.66 (-1.26, -0.06)*	-0.04 (-0.09, 0.00)	-0.07 (-0.13, -0.01)*
25(OH)D_2					
Continuous	-0.11 (-0.14, -0.08)*	-0.07 (-0.09, -0.05)*	-0.08 (-0.14, -0.01)*	-0.01 (-0.01, -0.00)*	0.02 (0.02, 0.03)*
Q1	Reference	Reference	Reference	Reference	Reference
Q2	-0.07 (-0.34, 0.19)*	-0.01 (-0.17, 0.15)	-0.40 (-0.99, 0.19)	-0.04 (-0.08, 0.01)	0.00 (-0.06, 0.07)
Q3	-0.63 (-0.89, -0.36)*	-0.33 (-0.49, -0.16)*	-0.86 (-1.46, -0.27)*	-0.08 (-0.12, -0.03)*	0.10 (0.03, 0.16)*
Q4	-0.86 (-1.13, -0.60)*	-0.52 (-0.69, -0.36)*	-0.50 (-1.09, 0.10)	-0.06 (-0.10, -0.01)*	0.18 (0.12, 0.25)*
25(OH)D					
Continuous	-0.01 (-0.02, 0.01)	-0.01 (-0.02, -0.00)*	-0.04 (-0.07, -0.02)*	-0.00 (-0.01, -0.00)*	0.00 (-0.00, 0.00)
Q1	Reference	Reference	Reference	Reference	Reference
Q2	-0.02 (-0.28, 0.25)	-0.08 (-0.24, 0.09)	0.28 (-0.31, 0.87)	0.02 (-0.02, 0.07)	0.04 (-0.03, 0.10)
Q3	0.12 (-0.14, 0.38)	0.02 (-0.14, 0.18)	-0.45 (-1.03, 0.12)	-0.02 (-0.07, 0.02)	-0.03 (-0.09, 0.03)
Q4	-0.06 (-0.34, 0.22)	-0.19 (-0.36, -0.02)*	-0.56 (-1.17, 0.06)	-0.04 (-0.09, 0.01)	0.00 (-0.06, 0.07)

Adjusted for alcohol intake, smoking status, physical activity, average monthly individual income, level of education, BMI, SBP, PP, TC, TG, HDL-C, LDL-C and family history of T2DM. *$P<0.05$. Abbreviation: Ln-, natural log - transformed; FPG, fasting plasm glucose; HbA1c, glycosylated hemoglobin; INS, insulin; *OR*, odds ratio; 95%*CI*: 95% confidence interval; Q1, the first/ lowest quartile; Q2, the second quartile; Q3, the third quartile; Q4, the fourth/highest quartile.

Supplement Table 3 Associations of 25(OH)D_3, 25(OH)D_2, total 25(OH)D (ng/ml) levels with IFG and T2DM in subgroups

Groups	Logistic regression, *OR*s (95%*CI*s)		
	25(OH)D_3	25(OH)D_2	25(OH)D
**IFG**			
Age<60y	0.98 (0.96, 1.00)	0.85 (0.80, 0.90)*	0.96 (0.94, 0.98)*
Age≥60y	0.98 (0.97, 1.00)	0.84 (0.80, 0.89)*	0.97 (0.95, 0.98)*
Male	0.97 (0.95, 0.99)*	0.82 (0.77, 0.88)*	0.95 (0.93, 0.97)*
Female	0.99 (0.97, 1.01)	0.86 (0.82, 0.90)*	0.98 (0.96, 0.99)*
BMI<24kg/m^2	0.98 (0.96, 1.00)*	0.82 (0.77, 0.86)*	0.96 (0.95, 0.98)*
BMI≥24kg/m^2	0.99 (0.97, 1.01)	0.87 (0.83, 0.92)*	0.97 (0.95, 0.99)*
No Hypertension	0.99 (0.98, 1.01)	0.85 (0.81, 0.89)*	0.97 (0.96, 0.99)*
Hypertension	0.97 (0.94, 1.00)*	0.83 (0.77, 0.90)*	0.95 (0.92, 0.97)*
No Dyslipidemia	0.99 (0.97, 1.00)	0.85 (0.81, 0.89)*	0.97 (0.96, 0.99)*
Dyslipidemia	0.98 (0.95, 1.00)	0.83 (0.78, 0.88)*	0.95 (0.93, 0.98)*
**T2DM**			
Age<60y	0.99 (0.97, 1.02)	0.83 (0.78, 0.88)*	0.97 (0.95, 0.99)*
Age≥60y	0.99 (0.97, 1.01)	0.85 (0.81, 0.90)*	0.97 (0.96, 0.99)*
Male	0.98 (0.96, 1.01)	0.82 (0.77, 0.88)*	0.96 (0.94, 0.98)*
Female	1.00 (0.98, 1.02)	0.86 (0.82, 0.91)*	0.98 (0.97, 1.00)
BMI<24kg/m^2	0.98 (0.95, 1.00)*	0.77 (0.72, 0.83)*	0.95 (0.94, 0.97)*
BMI≥24kg/m^2	1.01 (0.99, 1.03)	0.89 (0.84, 0.93)*	0.99 (0.97, 1.01)
No Hypertension	0.99 (0.98, 1.01)	0.86 (0.82, 0.90)*	0.98 (0.96, 0.99)*
Hypertension	0.99 (0.96, 1.02)	0.81 (0.75, 0.88)*	0.97 (0.94, 0.99)*
No Dyslipidemia	1.00 (0.98, 1.02)	0.86 (0.81, 0.91)*	0.98 (0.97, 1.00)
Dyslipidemia	0.99 (0.96, 1.01)	0.82 (0.78, 0.87)*	0.96 (0.94, 0.98)*

Adjusted for smoking status, alcohol intake, physical activity, average monthly individual income, level of education, SBP, PP, TC, TG, HDL-C, LDL-C and family history of T2DM. *P<0.05. Abbreviations: IFG, impaired fasting glucose; T2DM, type 2 diabetes mellitus; *OR*, odds ratio; 95%*CI*: 95% confidence interval.

Supplementary Table 4 The associations between vitamin D and lipid profile

	Adjusted β coefficients (95% *CIs*)			
	TC	TG	LDL-C	HDL-C
**IFG**				
**Male**				
25(OH)D_3	-0.0013 (-0.0092, 0.0066)	-0.0061 (-0.0172, 0.0049)	0.0000 (-0.0072, 0.0071)	0.0015 (-0.0019, 0.0050)
25(OH)D_2	-0.0171 (-0.0333, -0.0008)*	0.0118 (-0.0285, 0.0521)	-0.0181 (-0.0334, -0.0027)*	0.0022 (-0.0051, 0.0095)
25(OH)D	-0.0037 (-0.0101, 0.0027)	-0.0026 (-0.0140, 0.0088)	-0.0029 (-0.0091, 0.0033)	0.0015 (-0.0015, 0.0044)
**Female**				
25(OH)D_3	-0.0084 (-0.0159, -0.0008)*	-0.0128 (-0.0208, -0.0047)*	0.0006 (-0.0064, 0.0076)	0.0002 (-0.0028, 0.0031)
25(OH)D_2	-0.0334 (-0.0511, -0.0157)*	-0.0185 (-0.0363, -0.0007)*	-0.0253 (-0.0412, -0.0094)*	-0.0016 (-0.0094, 0.0061)
25(OH)D	-0.0109 (-0.0172, -0.0046)*	-0.0121 (-0.0188, -0.0054)*	-0.0031 (-0.0091, 0.0029)	-0.0001 (-0.0027, 0.0025)
**T2DM**				
**Male**				
25(OH)D_3	-0.0034 (-0.0115, 0.0047)	-0.0198 (-0.0327, -0.0069)*	0.0016 (-0.0060, 0.0091)	0.0042 (0.0010, 0.0074)*
25(OH)D_2	-0.0155 (-0.0322, 0.0011)	-0.0064 (-0.0490, 0.0362)	-0.0140 (-0.0294, 0.0013)	0.0069 (-0.0001, 0.0138)
25(OH)D	-0.0049 (-0.0115, 0.0016)	-0.0150 (-0.0282, -0.0018)*	-0.0012 (-0.0075, 0.0051)	0.0041 (0.0014, 0.0068)*
**Female**				
25(OH)D_3	-0.0077 (-0.0166, 0.0011)	-0.0023 (-0.0147, 0.0100)	-0.0088 (-0.0167, -0.0010)*	0.0007 (-0.0023, 0.0036)
25(OH)D_2	-0.0269 (-0.0456, -0.0082)*	-0.0324 (-0.0553, -0.0095)*	-0.0082 (-0.0243, 0.0080)	0.0048 (-0.0026, 0.0123)
25(OH)D	-0.0093 (-0.0167, -0.0019)*	-0.0062 (-0.0161, 0.0036)	-0.0074 (-0.0139, -0.0010)*	0.0012 (-0.0013, 0.0036)

Adjusted for alcohol intake, smoking status, physical activity, average monthly individual income, level of education, BMI, SBP, PP, and family history of T2DM. *$P<0.05$.

Abbreviation: TC, total cholesterol; TG, triglyceride; LDL-C, low - density lipoprotein cholesterol; HDL-C, high - density lipoprotein cholesterol; 95%*CI*: 95% confidence interval.

Supplement Table 5 The associations between testosterone and T2DM in premenopause and post-menopause females

T2DM	Logistic regression, *OR*s (95%*CI*s)	
	Premenopause females	Post-menopause females
Model 1	1.449 (1.031, 2.036)*	1.547 (1.332, 1.797)*
Model 2	1.512 (1.061, 2.154)*	1.534 (1.319, 1.784)*
Model 3	1.651 (1.112, 2.453)*	1.613 (1.362, 1.910)*

Model 1: no adjust. Model 2: adjusted for smoking status, alcohol intake, physical activity, average monthly individual income and level of education. Model 3: model 2+ BMI, SBP, PP, TC, TG, HDL-C, LDL-C and family history of T2DM. *P<0.05. Abbreviations: T2DM, type 2 diabetes mellitus; *OR*, odds ratio; 95%*CI*: 95% confidence interval.

Supplementary Table 6 Mediation analysis of the relationships between 25(OH)D_3, 25(OH)D_2 or 25(OH)D and IFG or T2DM by TC or TG in males and females

Parameters	TC		TG	
	**Male**	**Female**	**Male**	**Female**
**IFG**				
**25(OH)D_3**				
Total effect	-0.0314 (-0.0545, -0.0080)*	-0.0130 (-0.0305, -0.0052)*	-0.0314 (-0.0545, -0.0080)*	-0.0130 (-0.0305, -0.0052)*
Indirect effect-path *ab*	-0.0006 (-0.0043, 0.0028)	-0.0030 (-0.0065, -0.0005)*	-0.0009 (-0.0039, 0.0004)	-0.0017 (-0.0044, -0.0002)*
Path *a*	-0.0013 (-0.0092, 0.0066)	-0.0084 (-0.0159, -0.0008)*	-0.0061 (-0.0172, 0.0049)	-0.0128 (-0.0208, -0.0047)*
Path *b*	0.4142 (0.2025, 0.6260)*	0.3539 (0.2093, 0.4986)*	0.1390 (-0.0233, 0.3014)	0.1362 (0.0155, 0.2569)*
Direct effect-path c'	-0.0318 (-0.0533, -0.0103)*	-0.0102 (-0.0286, 0.0083)	-0.0305 (-0.0519, -0.0092)*	-0.0110 (-0.0293, 0.0074)
PE*	_	_b	_	_b
**25(OH)D_2**				
Total effect	-0.1951 (-0.2734, -0.1372)*	-0.1643 (-0.2211, -0.1153)*	-0.1951 (-0.2734, -0.1372)*	-0.1643 (-0.2211, -0.1153)*
Indirect effect-path *ab*	-0.0065 (-0.0156, -0.0007)*	-0.0105 (-0.0202, -0.0042)*	0.0021 (-0.0031, 0.0141)	-0.0023 (-0.0075, -0.0001)*
Path *a*	-0.0171 (-0.0333, -0.0008)*	-0.0334 (-0.0511, -0.0157)*	0.0118 (-0.0285, 0.0521)	-0.0185 (-0.0363, -0.0007)*
Path *b*	0.3809 (0.1626, 0.5993)*	0.3151 (0.1674, 0.4629)*	0.1744 (0.0030, 0.3458)*	0.1223 (-0.0003, 0.2450)
Direct effect-path c'	-0.1906 (-0.2514, -0.1298)*	-0.1532 (-0.2018, -0.1046)*	-0.1935 (-0.2540, -0.1330)*	-0.1620 (-0.2104, -0.1135)*
PE*	3.33%	6.39%	_	_
**25(OH)D**				
Total effect	-0.0493 (-0.0727, -0.0314)*	-0.0312 (-0.0486, -0.0157)*	-0.0493 (-0.0727, -0.0314)*	-0.0312 (-0.0486, -0.0157)*
Indirect effect-path *ab*	-0.0015 (-0.0051, 0.0010)	-0.0037 (-0.0069, -0.0015)*	-0.0004 (-0.0030, 0.0012)	-0.0015 (-0.0038, -0.0001)*
Path *a*	-0.0037 (-0.0101, 0.0027)	-0.0109 (-0.0172, -0.0046)*	-0.0026 (-0.0140, 0.0088)	-0.0121 (-0.0188, -0.0054)*

Path *b*	0.4067 (0.1923, 0.6210)*	0.3388 (0.1933, 0.4844)*	0.1449 (-0.0209, 0.3107)	0.1231 (0.0021, 0.2442)*
Direct effect-path c'	-0.0490 (-0.0683, -0.0297)*	-0.0278 (-0.0442, -0.0114)*	-0.0488 (-0.0679, -0.0296)*	-0.0295 (-0.0458, -0.0132)*
PE*	_	11.86%	_	4.81%
**T2DM**				
**25(OH)D_3**				
Total effect	-0.0192 (-0.0417, 0.0040)	-0.0019 (-0.0204, 0.0174)	-0.0192 (-0.0417, 0.0040)	-0.0019 (-0.0204, 0.0174)
Indirect effect-path *ab*	-0.0008 (-0.0040, 0.0009)	-0.0030 (-0.0070, 0.0003)	-0.0074 (-0.0146, -0.0024)*	-0.0010 (-0.0063, 0.0046)
Path *a*	-0.0034 (-0.0115, 0.0047)	-0.0077 (-0.0166, 0.0011)	-0.0198 (-0.0327, -0.0069)*	-0.0023 (-0.0147, 0.0100)
Path *b*	0.2409 (0.0425, 0.4394)*	0.3822 (0.2417, 0.5226)*	0.3738 (0.2120, 0.5355)*	0.4425 (0.3179, 0.5672)*
Direct effect-path c'	-0.0189 (-0.0414, 0.0036)	0.0015 (-0.0182, 0.0211)	-0.0141 (-0.0369, 0.0087)	-0.0007 (-0.0208, 0.0193)
PE*	_	_	_	_
**25(OH)D_2**				
Total effect	-0.2017 (-0.2960, -0.1324)*	-0.1786 (-0.2559, -0.1214)*	-0.2017 (-0.2960, -0.1324)*	-0.1786 (-0.2559, -0.1214)*
Indirect effect-path *ab*	-0.0034 (-0.0110, 0.0000)	-0.0097 (-0.0195, -0.0030)*	-0.0025 (-0.0190, 0.0142)	-0.0138 (-0.0258, -0.0041)*
Path *a*	-0.0155 (-0.0322, 0.0011)	-0.0269 (-0.0456, -0.0082)*	-0.0064 (-0.0490, 0.0362)	-0.0324 (-0.0553, -0.0095)*
Path *b*	0.2188 (0.0140, 0.4236)*	0.3611 (0.2171, 0.5051)*	0.3996 (0.2259, 0.5733)*	0.4270 (0.3003, 0.5538)*
Direct effect-path c'	-0.2006 (-0.2656, -0.1357)*	-0.1702 (-0.2223, -0.1181)*	-0.2022 (-0.2683, -0.1361)*	-0.1676 (-0.2198, -0.1154)*
PE*	_	5.43%	_	7.73%
**25(OH)D**				
Total effect	-0.0399 (-0.0619, -0.0213)*	-0.0238 (-0.0418, -0.0061)*	-0.0399 (-0.0619, -0.0213)*	-0.0238 (-0.0418, -0.0061)*
Indirect effect-path *ab*	-0.0012 (-0.0040, 0.0002)	-0.0035 (-0.0071, -0.0008)*	-0.0055 (-0.0122, -0.0009)*	-0.0027 (-0.0070, 0.0017)
Path *a*	-0.0049 (-0.0115, 0.0016)	-0.0093 (-0.0167, -0.0019)*	-0.0150 (-0.0282, -0.0018)*	-0.0062 (-0.0161, 0.0036)
Path *b*	0.2351 (0.0346, 0.4356)*	0.3712 (0.2302, 0.5123)*	0.3658 (0.2026, 0.5289)*	0.4381 (0.3136, 0.5627)*

Direct effect-path c'	-0.0394 (-0.0589, -0.0199)*	-0.0205 (-0.0374, -0.0036)*	-0.0369 (-0.0565, -0.0172)*	-0.0221 (-0.0393, -0.0049)*
PE*	_	14.71%	13.78%	_

PE*=Indirect effect/Total effect. b Fully mediated. Adjusted for alcohol intake, smoking status, physical activity, average monthly individual income, level of education, BMI, SBP, PP, and family history of T2DM. *P<0.05. Abbreviations: IFG, impaired fasting glucose; T2DM, type 2 diabetes mellitus; 95%CI: 95% confidence interval.

Supplementary Table 7 Mediation analysis of the relationships between 25(OH)D_3, 25(OH)D_2 or 25(OH)D and IFG or T2DM by LDL-C or HDL-C in males and females

Parameters	**LDL-C**		**HDL-C**	
	**Male**	**Female**	**Male**	**Female**
**IFG**				
**25(OH)D_3**				
Total effect	-0.0314 (-0.0545, -0.0080)*	-0.0130 (-0.0305, -0.0052)*	-0.0314 (-0.0545, -0.0080)*	-0.0130 (-0.0305, -0.0052)*
Indirect effect-path *ab*	0.0000 (-0.0025, 0.0025)	0.0002 (-0.0021, 0.0028)	-0.0001 (-0.0020, 0.0009)	0.0000 (-0.0010, 0.0005)
Path *a*	0.0000 (-0.0072, 0.0071)	0.0006 (-0.0064, 0.0076)	0.0015 (-0.0019, 0.0050)	0.0002 (-0.0028, 0.0031)
Path *b*	0.3066 (0.0797, 0.5334)*	0.3240 (0.1671, 0.4809)*	-0.0502 (-0.5506, 0.4502)	-0.0982 (-0.4649, 0.2685)
Direct effect-path c'	-0.0319 (-0.0533, -0.0106)*	-0.0134 (-0.0319, 0.0050)	-0.0313 (-0.0525, -0.0101)*	-0.0129 (-0.0312, 0.0053)
PE*	_	_	_	_
**25(OH)D_2**				
Total effect	-0.1951 (-0.2734, -0.1372)*	-0.1643 (-0.2211, -0.1153)*	-0.1951 (-0.2734, -0.1372)*	-0.1643 (-0.2211, -0.1153)*
Indirect effect-path *ab*	-0.0046 (-0.0132, -0.0001)*	-0.0072 (-0.0148, -0.0021)*	-0.0001 (-0.0035, 0.0018)	0.0002 (-0.0009, 0.0036)
Path *a*	-0.0181 (-0.0334, -0.0027)*	-0.0253 (-0.0412, -0.0094)*	0.0022 (-0.0051, 0.0095)	-0.0016 (-0.0094, 0.0061)
Path *b*	0.2563 (0.0224, 0.4903)*	0.2835 (0.1229, 0.4441)*	-0.0292 (-0.5484, 0.4899)	-0.1303 (-0.5076, 0.2471)

Direct effect-path c'	-0.1921 (-0.2527, -0.1315)*	-0.1579 (-0.2065, -0.1093)*	-0.1950 (-0.2555, -0.1345)*	-0.1647 (-0.2133, -0.1162)*
PE*	2.36%	4.38%	_	_
**25(OH)D**				
Total effect	-0.0493 (-0.0727, -0.0314)*	-0.0312 (-0.0486, -0.0157)*	-0.0493 (-0.0727, -0.0314)*	-0.0312 (-0.0486, -0.0157)*
Indirect effect-path *ab*	-0.0009 (-0.0037, 0.0008)	-0.0010 (-0.0035, 0.0008)	0.0000 (-0.0016, 0.0009)	0.0000 (-0.0005, 0.0008)
Path *a*	-0.0029 (-0.0091, 0.0033)	-0.0031 (-0.0091, 0.0029)	0.0015 (-0.0015, 0.0044)	-0.0001 (-0.0027, 0.0025)
Path *b*	0.2967 (0.0669, 0.5265)*	0.3188 (0.1608, 0.4767)*	-0.0284 (-0.5370, 0.4801)	-0.1013 (-0.4704, 0.2678)
Direct effect-path c'	-0.0493 (-0.0686, -0.0301)*	-0.0307 (-0.0471, -0.0144)*	-0.0493 (-0.0684, -0.0302)*	-0.0312 (-0.0474, -0.0150)*
PE*	_	_	_	_
**T2DM**				
**25(OH)D_3**				
Total effect	-0.0192 (-0.0417, 0.0040)	-0.0019 (-0.0204, 0.0174)	-0.0192 (-0.0417, 0.0040)	-0.0019 (-0.0204, 0.0174)
Indirect effect-path *ab*	-0.0003 (-0.0029, 0.0009)	-0.0009 (-0.0035, 0.0002)	-0.0045 (-0.0099, -0.0010)*	-0.0004 (-0.0028, 0.0016)
Path *a*	0.0016 (-0.0060, 0.0091)	-0.0088 (-0.0167, -0.0010)*	0.0042 (0.0010, 0.0074)*	0.0007 (-0.0023, 0.0036)
Path *b*	-0.1860 (-0.4037, 0.0317)	0.1073 (-0.0426, 0.2572)	-1.0722 (-1.6477, -0.4966)*	-0.6637 (-1.0647, -0.2628)*
Direct effect-path c'	-0.0190 (-0.0413, 0.0033)	-0.0009 (-0.0203, 0.0185)	-0.0152 (-0.0378, 0.0074)	-0.0017 (-0.0211, 0.0178)
PE*	_	_	_	_
**25(OH)D_2**				
Total effect	-0.2017 (-0.2960, -0.1324)*	-0.1786 (-0.2559, -0.1214)*	-0.2017 (-0.2960, -0.1324)*	-0.1786 (-0.2559, -0.1214)*
Indirect effect-path *ab*	0.0034 (0.0000, 0.0106)*	-0.0008 (-0.0048, 0.0006)	-0.0066 (-0.0178, -0.0001)*	-0.0032 (-0.0101, 0.0010)
Path *a*	-0.0140 (-0.0294, 0.0013)	-0.0082 (-0.0243, 0.0080)	0.0069 (-0.0001, 0.0138)	0.0048 (-0.0026, 0.0123)
Path *b*	-0.2447 (-0.4727, -0.0168)*	0.0990 (-0.0543, 0.2523)	-0.9651 (-1.5603, -0.3699)*	-0.6512 (-1.0659, -0.2365)*
Direct effect-path c'	-0.2063 (-0.2716, -0.1410)*	-0.1781 (-0.2299, -0.1263)*	.	-0.1780 (-0.2299, -0.1261)*

PE*	_	_	_	_
**25(OH)D**				
Total effect	-0.0399 (-0.0619, -0.0213)*	-0.0238 (-0.0418, -0.0061)*	-0.0399 (-0.0619, -0.0213)*	-0.0238 (-0.0418, -0.0061)*
Indirect effect-path *ab*	0.0002 (-0.0010, 0.0022)	-0.0007 (-0.0028, 0.0003)	-0.0040 (-0.0089, -0.0012)*	-0.0008 (-0.0031, 0.0007)
Path *a*	-0.0012 (-0.0075, 0.0051)	-0.0074 (-0.0139, -0.0010)*	0.0041 (0.0014, 0.0068)*	0.0012 (-0.0013, 0.0036)
Path *b*	-0.1968 (-0.4181, 0.0245)	0.0939 (-0.0565, 0.2443)	-0.9941 (-1.5760, -0.4122)*	-0.6567 (-1.0598, -0.2537)*
Direct effect-path c'	-0.0402 (-0.0596, -0.0207)*	-0.0231 (-0.0399, -0.0064)*	-0.0364 (-0.0560, -0.0167)*	-0.0234 (-0.0402, -0.0066)*
PE*	_	_	10.03%	_

PE*=Indirect effect/Total effect. b Fully mediated. Adjusted for alcohol intake, smoking status, physical activity, average monthly individual income, level of education, BMI, SBP, PP, and family history of T2DM. *P<0.05. Abbreviations: IFG, impaired fasting glucose; T2DM, type 2 diabetes mellitus; 95%CI: 95% confidence interval.

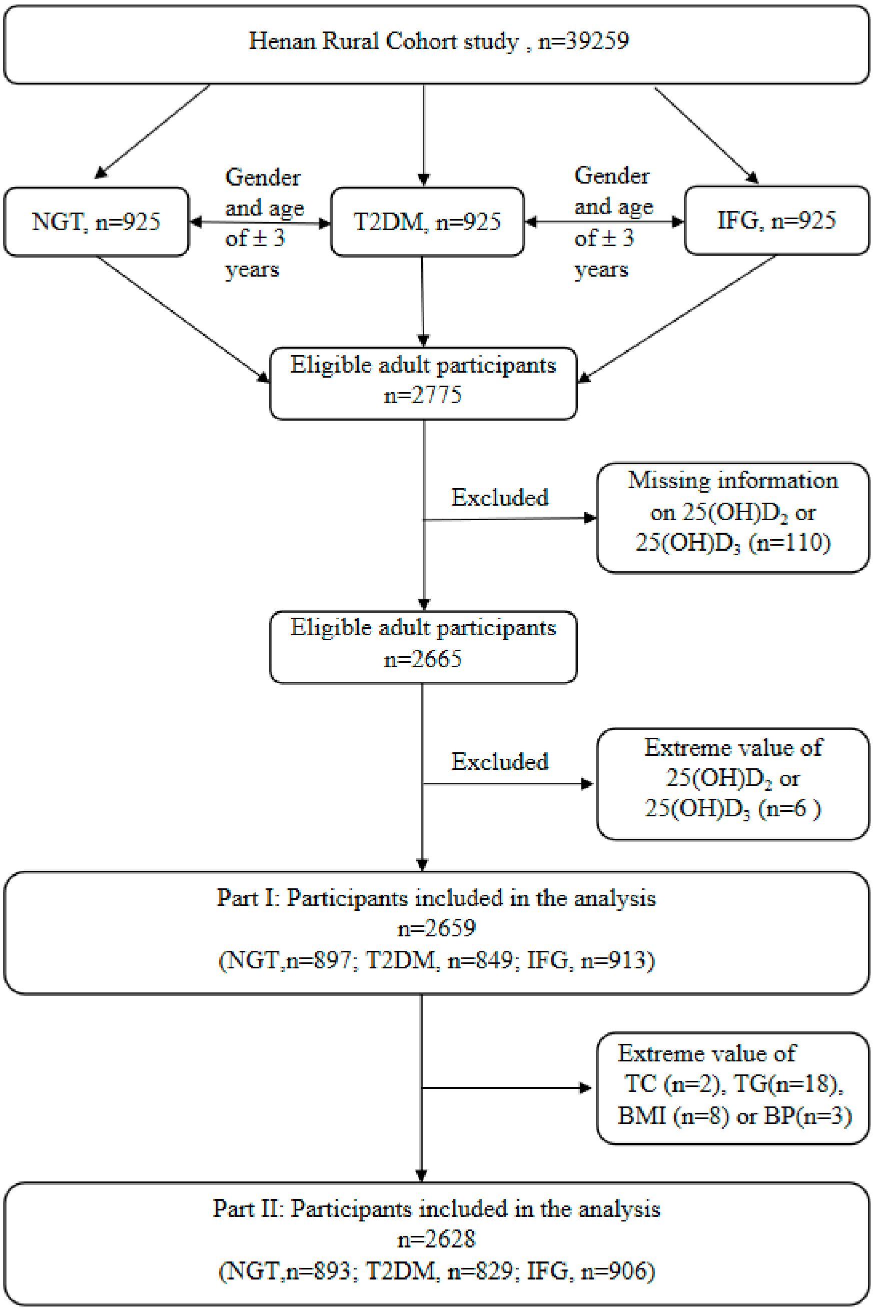

Supplement Fig. 1 The flowchart of the inclusion and exclusion of participants.

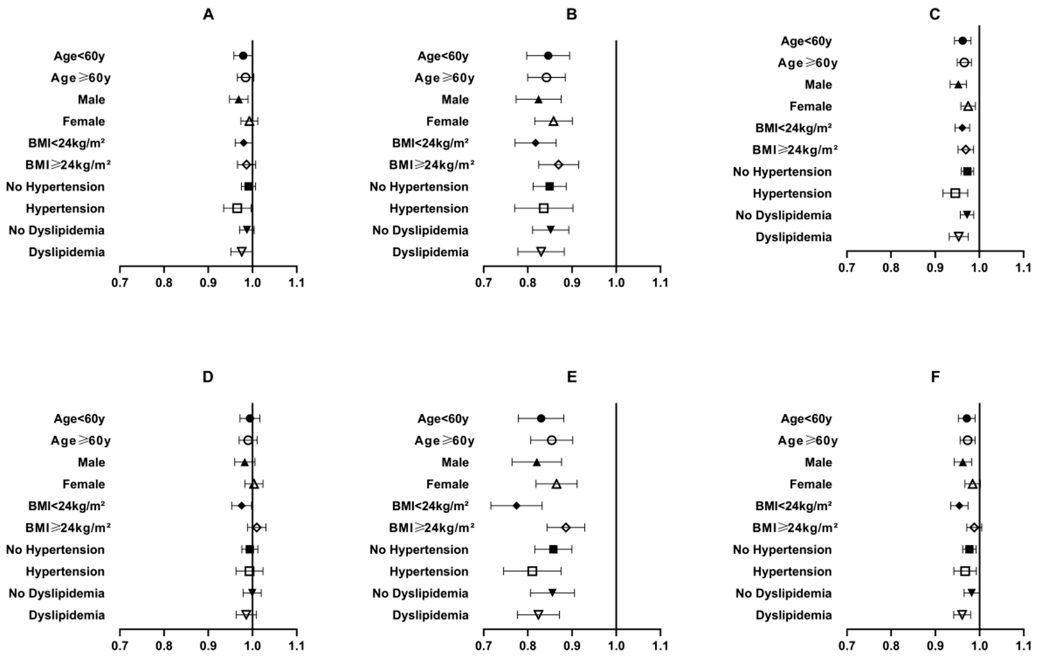

Supplement Fig. 2 The associations of 25(OH)D_3, 25(OH)D_2 and total 25(OH)D on IFG and T2DM in subgroups. *OR*s and 95%*CI*s for stratification analysis by age (< 60 years or ≥ 60 years), gender (male or female), BMI (< 24 kg/m^2 or ≥ 24 kg/m^2), hypertension (no or yes), dyslipidemia (no or yes) for 25(OH)D_3, 25(OH)D_2, total 25(OH)D on IFG and T2DM compared with NGT, respectively. "A", "B", "C" represent the associations of 25(OH)D_3, 25(OH)D_2 and total 25(OH)D on IFG; "D", "E", "F" represent the associations of 25(OH)D_3, 25(OH)D_2 and total 25(OH)D on T2DM; adjusted for smoking status, alcohol intake, physical activity, average monthly individual income, level of education, SBP, PP, TC, TG, HDL-C, LDL-C and family history of T2DM.
